# Feasibility of a Mobile Health App for Routine Outcome Monitoring and Feedback in SMART Recovery Mutual Support Groups: Stage 1 Mixed Methods Pilot Study

**DOI:** 10.2196/25217

**Published:** 2021-10-06

**Authors:** Peter J Kelly, Alison K Beck, Frank P Deane, Briony Larance, Amanda L Baker, Leanne Hides, Victoria Manning, Anthony Shakeshaft, Joanne Neale, John F Kelly, Christopher Oldmeadow, Andrew Searles, Kerrin Palazzi, Kenny Lawson, Carla Treloar, Rebecca M Gray, Angela Argent, Ryan McGlaughlin

**Affiliations:** 1 School of Psychology Faculty of Arts, Social Sciences and Humanities University of Wollongong Wollongong Australia; 2 Illawarra Health and Medical Research Institute University of Wollongong Wollongong Australia; 3 School of Medicine and Public Health University of Newcastle Newcastle Australia; 4 Centre for Youth Substance Abuse Research, Lives Lived Well Group School of Psychology University of Queensland Brisbane St Lucia Australia; 5 Eastern Health Clinical School, Faculty of Medicine Nursing and Health Sciences Monash University Box Hill Australia; 6 National Drug and Alcohol Research Centre University of New South Wales Sydney Australia; 7 Institute of Psychiatry Psychology and Neuroscience King’s College London London United Kingdom; 8 Harvard Medical School Harvard University Boston, MA United States; 9 Clinical Research Design, IT and Statistical Support Unit Hunter Medical Research Institute New Lambton Australia; 10 Hunter Medical Research Institute Health Research Economics Hunter Medical Research Institute New Lambton Australia; 11 Centre for Social Research in Health Faculty of Arts and Social Sciences University of New South Wales Sydney Australia; 12 SMART Recovery Australia Sydney Australia

**Keywords:** mHealth, SMART Recovery, mutual support group, mutual aid, routine outcome monitoring, treatment progress feedback, addiction, mobile phone

## Abstract

**Background:**

Mutual support groups are an important source of long-term help for people impacted by addictive behaviors. Routine outcome monitoring (ROM) and feedback are yet to be implemented in these settings. SMART Recovery mutual support groups focus on self-empowerment and use evidence-based techniques (eg, motivational and behavioral strategies). Trained facilitators lead all SMART Recovery groups, providing an opportunity to implement ROM.

**Objective:**

The aim of this stage 1 pilot study is to explore the feasibility, acceptability, and preliminary outcomes of a novel, purpose-built mobile health ROM and feedback app (*SMART Track*) in mutual support groups coordinated by SMART Recovery Australia (SRAU) over 8 weeks.

**Methods:**

*SMART Track* was developed during phase 1 of this study using participatory design methods and an iterative development process. During phase 2, 72 SRAU group participants were recruited to a nonrandomized, prospective, single-arm trial of the *SMART Track* app. Four modes of data collection were used: ROM data directly entered by participants into the app; app data analytics captured by Amplitude Analytics (number of visits, number of unique users, visit duration, time of visit, and user retention); baseline, 2-, and 8-week follow-up assessments conducted through telephone; and qualitative telephone interviews with a convenience sample of study participants (20/72, 28%) and facilitators (n=8).

**Results:**

Of the 72 study participants, 68 (94%) created a *SMART Track* account, 64 (88%) used *SMART Track* at least once, and 42 (58%) used the app for more than 5 weeks. During week 1, 83% (60/72) of participants entered ROM data for one or more outcomes, decreasing to 31% (22/72) by the end of 8 weeks. The two main screens designed to provide personal feedback data (*Urges* screen and *Overall Progress* screen) were the most frequently visited sections of the app. Qualitative feedback from participants and facilitators supported the acceptability of *SMART Track* and the need for improved integration into the SRAU groups. Participants reported significant reductions between the baseline and 8- week scores on the Severity of Dependence Scale (mean difference 1.93, SD 3.02; 95% CI 1.12-2.73) and the Kessler Psychological Distress Scale-10 (mean difference 3.96, SD 8.31; 95% CI 1.75-6.17), but no change on the Substance Use Recovery Evaluator (mean difference 0.11, SD 7.97; 95% CI –2.02 to 2.24) was reported.

**Conclusions:**

Findings support the feasibility, acceptability, and utility of *SMART Track*. Given that sustained engagement with mobile health apps is notoriously difficult to achieve, our findings are promising. *SMART Track* offers a potential solution for ROM and personal feedback, particularly for people with substance use disorders who attend mutual support groups.

**Trial Registration:**

Australian New Zealand Clinical Trials Registry ACTRN12619000686101; https://anzctr.org.au/Trial/Registration/TrialReview.aspx?id=377336

**International Registered Report Identifier (IRRID):**

RR2-10.2196/15113

## Introduction

### Background

Routine outcome monitoring (ROM) is central to evidence-based health care for a range of chronic conditions [1], including addictive behaviors [2,3]. ROM is central to understanding, evaluating, and improving service delivery [4-6]. A range of clinical benefits have been identified [7-9], particularly for those people identified as *not on track* early in the course of treatment [10,11]. Emerging evidence suggests that providing clients with tailored feedback may be central to demonstrated improvements in client outcomes [12].

To date, ROM and feedback have been implemented in a range of mental health [13] and addiction [14,15] treatment settings but not in mutual support groups. Mutual support groups offer an important source of fee-free, accessible support to people experiencing a range of addictive behaviors. Mutual support is particularly important for people experiencing addictive behaviors, given the often long-term and nonlinear process of recovery [16]. Mutual support groups may be attended before, during, after, or in lieu of engagement with formal treatment services, providing the potential for continuity across the recovery process. Although accumulating evidence highlights the importance and benefits of participating in mutual support [17-21], a major limitation is the lack of systematically collected data evaluating the outcomes. Unlike other clinically endorsed [2,3] models of mutual support for addictive behaviors (eg, 12-step approaches), SMART Recovery groups use a trained facilitator. This provides a unique opportunity to work with group facilitators to embed ROM and personal feedback as a standard component of the groups.

Integrating ROM and tailored feedback into routine service provision is not without challenges [22,23]. Common barriers include the *time burden* associated with completing, scoring, interpreting, or discussing outcome assessments [22,24], as well as skepticism regarding the perceived relevance of the outcomes assessed and feedback generated [25,26]. Additional limitations include the traditionally clinician-centric nature of ROM (see studies by Carlier and van Eden [7] and Thompson et al [13] for a discussion and studies by Lambert et al [8], Goodman et al [12], and Burgess et al [27] for common instruments) and accompanying feedback [28,29]. Improved acknowledgment of the client perspective during assessment [30] and greater client involvement in the feedback process [31] are both important clinical and research priorities.

The idea of using technology to track progress within health care settings is not new, but current approaches are limited [32]. Unlike other health information technology approaches (eg, web-based platforms), mobile health (mHealth [33]) apps offer a quick, easy, interactive, and engaging platform for tracking and accessing information about health and health-related behaviors [34]. A key benefit of mHealth apps is their ability to provide timely, individualized feedback [35]. Given the ubiquity of smartphone ownership [36,37], smartphone apps can engage individuals in real time and in their natural environment and by offering moment-to-moment support as needed [38]. Indeed, a recent systematic review of digital support services highlighted that their *on-demand* nature is a key benefit [39].

Although not specifically designed for the purposes of ROM and feedback, mHealth apps with the capability to track a variety of health behaviors, conditions, or outcomes [40-45], including alcohol consumption, substance use, and other addictive behaviors [39,44-52], have been developed. However, a key limitation is the ever-increasing gap between the availability of mHealth apps and their scientific validation [40,52-55]. Moreover, the level of end-user involvement throughout the development process is often unclear. This is important because inadequate consideration of the needs and preferences of the end user has been implicated in mHealth attrition [56-58]. Accordingly, we worked alongside end users to develop a purpose-built mHealth app for ROM and feedback in SMART Recovery Australia (SRAU) mutual support groups (*SMART Track*), which was then evaluated in this study.

### Objective

The aim of this stage 1 nonrandomized, single-arm pilot study is to explore the feasibility, acceptability, and preliminary outcomes of a novel mHealth ROM and feedback app (*SMART Track*) in mutual support groups coordinated by SRAU.

## Methods

### Overview

Approval was granted by the University of Wollongong and Illawarra Shoalhaven Local Health District Health and Medical Human Research Ethics Committee (2018/099; HREC/18/WGONG/34). The study was registered with the Australian New Zealand Clinical Trials Registry (ACTRN12619000686101), and a protocol was published [59]. The reporting of this study follows the CONSORT (Consolidated Standards of Reporting Trials)-EHEALTH checklist [60].

### Setting

Participants were recruited from the SMART Recovery groups registered with SRAU. Detailed accounts of SMART Recovery groups have been published [61]. Briefly, SMART Recovery groups originated in the United States and are now available across 23 countries. They offer support for people experiencing a range of addictive behaviors, including substance- and non–substance-related behaviors. SMART Recovery groups focus on self-empowerment and use evidence-based techniques (eg, cognitive behavioral therapy and motivational interviewing) [62]. These groups are held in a variety of community, inpatient, outpatient, residential rehabilitation, and clinical health settings. Online support groups are also available.

**Figure 1:**
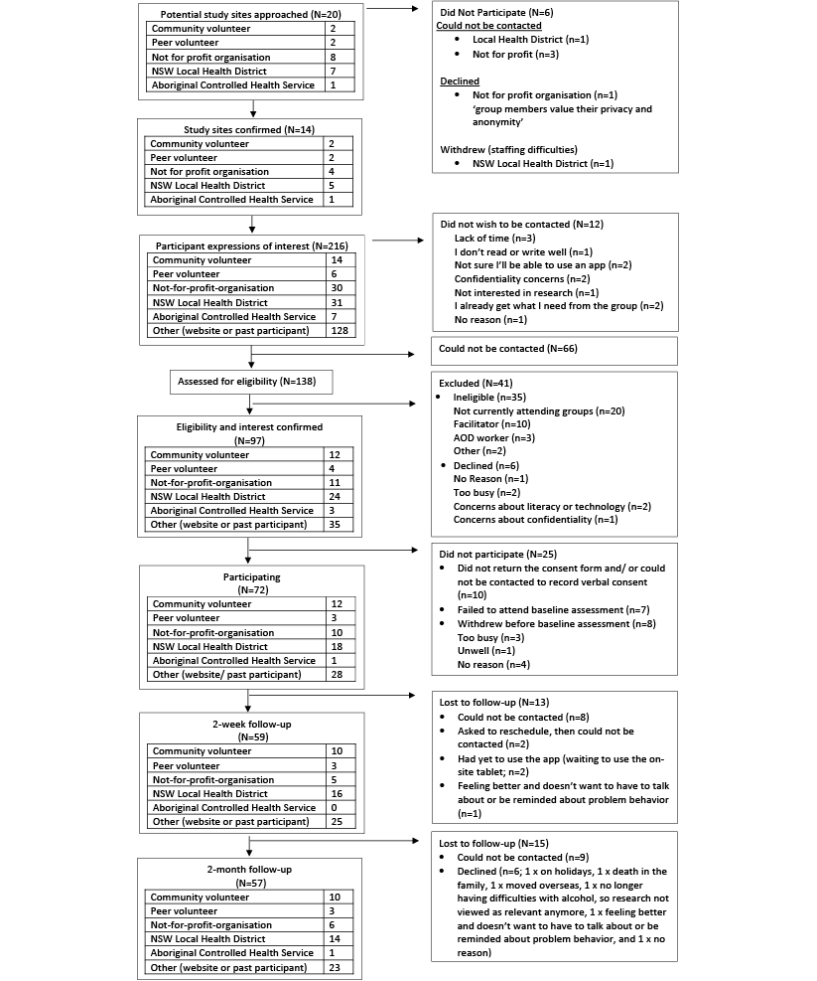
CONSORT (Consolidated Standards of Reporting Trials) flow diagram depicting the number of participants referred, lost, and retained according to the referral source. AOD: alcohol and other drugs; NSW: New South Wales.

We invited 20 sites in New South Wales, Australia, to participate in this study, and 14 (70%) agreed (Figure 1). To enhance generalizability, the invited sites were selected to reflect a range of geographical locations and service providers. We sought to recruit 100 study participants. A sample of this size was selected to allow estimation of the recruitment rate and 95% CI with a margin of error of no more than 7%.

### Participants

Participants were eligible if they were aged at least 18 years, were currently participating in SRAU groups (either face-to-face or online), had (or were willing to obtain) an email address, and comprehended English at a level sufficient to complete the study requirements. Participants were eligible irrespective of self-reported computer or smartphone literacy, and they did not have to own a smartphone. The study sites were provided with an Android (Samsung Galaxy Tab A) tablet for on-site participant use. No restrictions were placed on concomitant care or the frequency or duration of SMART Recovery group participation. The only exclusion criterion was inability or unwillingness to provide informed consent.

### Recruitment

A group facilitator or member of the research team provided potential participants with standardized written and verbal information at the beginning of the SMART Recovery group session. Potential participants were asked to provide their preferred contact details, and they were contacted directly by a member of the research team. To avoid any potential coercion or desirability bias arising from the working relationship between facilitators and participants, the researcher (not the facilitator) was responsible for confirming participant interest and seeking informed consent. To boost accrual, during the final month of recruitment, a web-based expression-of-interest form (displayed prominently on the SRAU website) was introduced. Potential participants could contact the research team directly through email, phone, or the web-based expression-of-interest form. All participants provided verbal or written informed consent. The participants were reimbursed (Aus $30 [US $22.02] supermarket voucher) for their time, travel, and effort associated with each interview: baseline and 8-week assessments, as well as qualitative interviews (up to a total of Aus $90 [US $66.06]).

### SMART Track: ROM and Feedback mHealth App

#### Development

##### Overview

The preparatory qualitative work [63] and development process [64] have been reported separately, and further details are available in the published protocol [59]. Briefly, three frameworks [56,65,66] informed the design, development, and content of *SMART Track*. Although each framework can be used in isolation, we chose to combine these approaches to ensure that app development was informed by a more comprehensive set of guidelines that included foci related to the end user (ie, *person*; *person-based* [56]); best practice recommendations for mHealth development (Behavioral Intervention Technology Model [65]); and a collaborative, iterative development process involving the research team, app developers, and participants (Integrate, Design, Assess, and Share Framework [66]). *SMART Track* is grounded in behavioral theory (Self-Determination Theory [67] and Social Control Theory [68]) and the guiding principles of SMART Recovery (self-management, mutual aid, and choice [69]). Behavioral strategies are drawn from the Behavior Change Taxonomy (self-monitoring, feedback, action planning, prompts or cues, and nonspecific reward [70]) and process motivators (choice or control, competence, context, curiosity, personalization, and reframing [66]). The agency contracted for app development and design was GHO, Sydney [71].

##### Beta-Testing

The initial beta version of the app was submitted to the Apple App Store and Google Play Store for approval in March 2019. The functionality of the app was initially tested with 3 members of the research team (beginning April 5, 2019). Several bugs were identified and fixed before the emended beta version was released (June 4, 2019) for further testing to a convenience sample comprising 40 members of the SRAU Research Advisory Committee, SRAU steering committee, and SMART Recovery board, as well as SMART Recovery facilitators. Further refinements were made in line with the feedback (bug fixes and minor amendments to functionality and content). The participant version of *SMART Track* was available in the Google Play Store (version 0.0.7) and Apple App Store (version 0.7) on July 15, 2019. *SMART Track* is freely available for Android [72] and Apple [73] devices.

##### Revisions and Updating

The time frame of the weekly period of ROM data collection was emended in July 2019 (from closing 24 hours after the nominated meeting began to closing 30 minutes after the nominated meeting began). This was to enable the next week of data collection to begin during the meeting such that the participants could set a new 7-day plan at the end of the meeting (rather than having to wait 24 hours). Cloud functions were updated in September 2019 to fix to participant reports (4/72, 6%) that they had not received the expected prompt from *SMART Track* to complete the ROM items.

#### Overview

##### Summary

The *SMART Track* app is designed for participants attending SMART Recovery groups (either face-to-face or online). *SMART Track* comprises core ROM and feedback functionality and several additional features to enhance engagement (resources, customizable supports, personal motivations, interactive urge log, and pop-up motivations and self-management strategies, as described below). The content is distributed across five main screens (Figure S1 of Multimedia Appendix 1).

##### ROM Domains and Items

Consistent with clinical guidelines [2,3] and published recommendations [7,31], *SMART Track* provides multidimensional assessment and feedback. The items included in the app are detailed in Table S1 of Multimedia Appendix 2 [74-83] as a function of target domain and assessment frequency. Further details are available in the published protocol [59]. Briefly, the participants were prompted each week to answer a set of questions, and their responses were used to provide tailored progress feedback.

##### Progress Feedback

Feedback consists of tailored visual and written feedback across eight domains (7-day plan, behavior of concern, effect of substance use, self-care, relationships, outlook on life, resources, and mental health; see Tables S2 and S3 of Multimedia Appendix 2 for the scoring algorithms).

##### Resources

The *Resources* screen is able to deliver a maximum of 10 pieces of content. This was distributed across seven self-management resources (including SMART Recovery resources) and three motivational stories (extracted with permission from the *Lives of Substance* website [84]). Content upload was managed by the research team using *WordPress* according to the schedule outlined in Table S4 of Multimedia Appendix 2.

##### Customizable Supports and Personal Motivations

Participants have the option of tailoring app content by uploading one or more contact numbers, support services or personal motivations for change (photo, audio, video, or text) into the *Me* section of the app.

##### Interactive Urge Log

In addition to tracking the number, frequency, and strength of urges, when the participant reports an urge, this interactive tool prompts them to manage their urges, log triggers, and reflect on how to maintain or improve effective urge self-management. The interactive urge log contains a range of urge management strategies or motivational content (Table S5 of Multimedia Appendix 2). The content was derived from SMART Recovery manuals [85,86] and transcripts of participants’ qualitative interviews [63] and presented to the participants in random order. The participants could also use the *Me* section of the app to enter their own personal strategies and motivations. Participant-entered content is always shown before prespecified content, and it is not accessible to other participants.

##### Pop-up Motivations and Self-management Strategies

The participants received *pop*-up messages when they opened the app for the first time each day (Table S6 of Multimedia Appendix 2). This content is derived from transcripts of qualitative interviews [63]. A combination of direct excerpts and emended content (modified for clarity) was used.

#### Implementation

##### Orientation

After completion of the baseline assessment, the researcher asked the participants to use *SMART Track* at least once a week to complete the ROM questions and enter their 7-day plan and use the other app functions *as needed*. This was reinforced in an introductory email, which also contained the Google Play Store and Apple App Store links to download the app. SMART Recovery facilitators were asked to prompt the participants at the beginning and end of each group session. No additional training or support was provided (outside of what may have been naturally provided by facilitators and peers as part of the group session). A *walk*-*through* is included in the app to orient participants to the app (Figure S1 of Multimedia Appendix 1).

##### Prompts and Reminders

The 7-day plan and ROM questions were linked to the day and time that the participant used *SMART Track* in their regular SMART Recovery group session. Tasks were set for 7 days after the meeting. The 7-day plan notifications were customizable. For each task, the participants elected whether and when to receive a reminder notification. A notification to complete the ROM questions was automatically sent 24 hours before the nominated group session. If the questions were not answered, additional reminders were sent 12 hours and again 30 minutes before the group session. The ROM notifications could not be *switched off* by the participant.

##### Privacy and Confidentiality

To allow participants to reset their password, *SMART Track* captures the email addresses of all end users. However, it is up to the participant to decide whether the email address they choose to use contains any element of personal information (eg, their name). Given the potential impact of privacy- and confidentiality-related concerns on participant engagement with *SMART Track*, a comprehensive privacy and confidentiality policy is available.

### Data Collection Procedures

#### Overview

**Figure 2:**
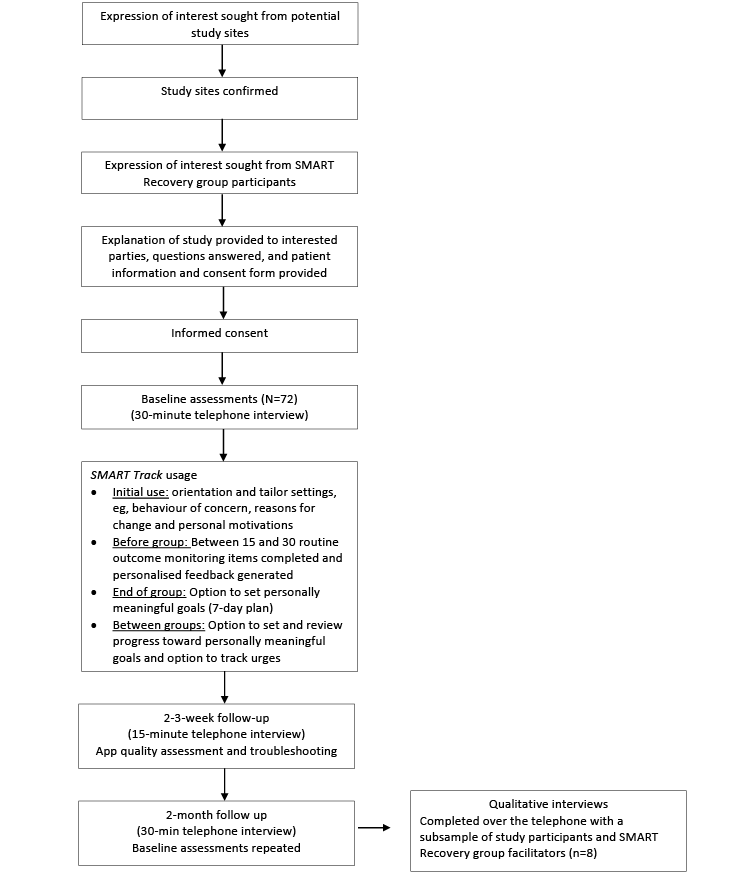
Flowchart of study activities.

The study activities are outlined in Figure 2. The four modes of data collection included (1) participant-completed ROM data collected through *SMART Track* (Table S1 of Multimedia Appendix 2); (2) app data analytics captured using Amplitude Analytics (Amplitude, Inc; number of visits, number of unique users, visit duration, time of visit, and user retention) [87]; (3) baseline, 2-week, and 8-week follow-up assessments conducted over the telephone by AKB; and (4) qualitative interviews with the study participants and group facilitators (conducted over the telephone by RMG). The primary and secondary objectives, measures, and indicator variables are summarized in Table 1.

**Table 1:** Primary and secondary objectives, measures, and indicators.

Objectives		Variables
**Primary objectives (measures and indicators)**		
	To explore the feasibility of using *SMART Track* as part of SMART Recovery groups	Proportion of eligible participants who consent to the study, create an account, and use SMART Track Proportion of missing data for each of the routine outcome monitoring items and instruments at each week of administration across the 8-week period of SMART Track use Engagement and use patterns indexed through data analytics captured daily across the data collection period Costs associated with developing SMART Track and maintaining the app until the completion of data collection
	To explore the acceptability of using *SMART Track* as part of SMART Recovery groups	Detailed qualitative feedback from SMART Recovery group members and facilitators to explore their experience of, and satisfaction with, SMART Track (8-week follow-up) Quality ratings as assessed by participant ratings of the end-user version [87] of the Mobile App Rating Scale [88] at 8-week follow-up Digital therapeutic alliance ratings as assessed by participant ratings of the Digital Working Alliance Inventory at 8-week follow-up
**Secondary objective (secondary end points)**		
	To provide preliminary evidence for participant-reported outcomes	Participant-reported progress across the 8-week period of app use in (1) substance dependence (Severity of Dependence Scale [89]), (2) addiction recovery (Substance Use Recovery Evaluator [74]), and (3) mental health (Kessler Psychological Distress Scale [75,90])

#### Key Measures and Assessment Instruments

##### Overview

The study measures and assessment instruments are detailed in the published protocol [59] and summarized in Table 2. Feasibility and acceptability were informed by data analytics captured using Amplitude Analytics (number of visits, number of unique users, visit duration, time of visit, and user retention) [87]; qualitative interviews; quality assessment conducted using the simplified, end-user version [88] of the Mobile App Rating Scale (MARS) [89] and the Digital Working Alliance Inventory (DWAI) [92]; and a cost analysis informed by a *cost capture template* [93-95] and an adapted version of the Client Service Receipt Inventory—*Generic* UK Mental Health [96]. Preliminary evidence for participant-reported outcomes after the use of *SMART Track* in conjunction with SMART Recovery groups was captured using the Severity of Dependence Scale (SDS) [90], Kessler Psychological Distress Scale-10 (K-10) [97], and the Substance Use Recovery Evaluator (SURE) [74].

**Table 2:** Schedule of data collection.

				Baseline		Daily		Weekly		2-week follow-up		8-week follow-up
**SMART Recovery participants**												
	***SMART Track* app**											
		Data analytics			✓^a^							
		ROM^b^ items^c^					✓					
	Demographics			✓								✓
	**NADA^d^ COMS^e^**											
		Severity of Dependence Scale	✓								✓	
		Drug and Alcohol Use	✓								✓	
		Kessler 10+	✓								✓	
		The World Health Organization Quality of Life 8	✓								✓	
		NSW^f^ Minimum Data Set items (living arrangements and income)	✓								✓	
		BTOM-C^g^ items on arrests	✓								✓	
		BTOM-C items on risky drug using practices	✓								✓	
	Substance Use Recovery Evaluator			✓								✓
	Client Services Receipt Inventory			✓								✓
	Mobile Application Rating Scale–User Version									✓		
	Digital Working Alliance Inventory									✓		✓
	Qualitative interview (n=20)											✓
**SMART Recovery facilitators**												
	Demographics											✓
	Mobile App Rating Scale											✓
	Qualitative interview (n=8)											✓

^a^Data collected.

^b^ROM: routine outcome monitoring.

^c^See Multimedia Appendix 2 (Table S1) for a detailed description of routine outcome monitoring items as a function of assessment domain and frequency of administration.

^d^NADA: Network of Alcohol and Other Drugs Agencies.

^e^COMS: Client Outcomes Management System.

^f^NSW: New South Wales.

^g^BTOM-C: Brief Treatment Outcome Measure—Concise.

##### Nested Qualitative Evaluation

Qualitative interviews were conducted by RMG after the 8-week period of app use to explore the experiences and opinions of participants with diverse engagement with *SMART Track*. The participants were sampled to reflect the diversity of their characteristics (gender and primary behavior of concern), referral source, and pattern of *SMART Track* use. An independent qualitative researcher (RMG) used a topic guide (Table S7 of Multimedia Appendix 2) to ask additional open-ended questions of a selection of participants (n=20) and facilitators (n=8). The participants and facilitators were sampled to reflect diversity in gender, geographical location, and (for participants only) behavior of concern. For the app users, this included the pathway to SMART Recovery groups (opening and warm-up), perceptions and experiences of app use, initiation circumstances for the app, motivation to join the trial and use the app, and suggestions for improvements. The facilitators were asked similar questions, but the focus was on their professional capacity rather than on their personal experience with apps. The interview started by eliciting information about how they came to be a facilitator, how the app was initiated with their group, their perceptions and experiences in implementing the app with the service users, their motivation levels related to the implementation of the app, and suggestions for improvements. All interviews were audio recorded and transcribed by a professional transcriber working under a confidentiality agreement.

### Analysis

#### Feasibility Indicators

##### Enrollment and Engagement

Data analytics were captured daily from the time the app was launched until the last participant completed their 8-week follow-up interview. The first participant was given the download details on July 15, 2019, and follow-up data collection was completed on December 2, 2019. Weekly summaries for the total number of unique users and the average number of visits per user were downloaded from Amplitude Analytics. Unique user codes were linked, and the number of weeks that each study participant used the app was calculated.

##### Use Patterns

To explore how the participants engaged with the various features of *SMART Track*, weekly analytics (total number of visits, total number of unique users, and total duration) for each of the *SMART Track* features (*Urge button*, *Urges* screen, *Resources* screen, *Me* screen, and *Overall Progress* screen) and the time of day that the app was used were downloaded from Amplitude Analytics and descriptive statistics calculated. Retention was characterized using weekly summaries from the *User Lifecycle* feature of Amplitude Analytics. This feature categorizes participants into the following mutually exclusive categories:

1. New users (used the app for the first time that week).
2. Current users (used the app at least once that week and at least once during the preceding week).
3. Resurrected users (used the app at least once during the week after being dormant during at least the previous week).
4. Dormant users (did not use the app that week but did use the app at least once during the preceding week).

##### Proportion of Missing ROM Data

ROM use (yes or no) for each week of the 8-week follow-up period was defined as participant entry of *SMART Track* data for at least one outcome domain (7-day plan, ROM questionnaire, and Urges). This was used to calculate the weekly proportion of participants who entered the data.

##### Research and Development Costs

An economic costing analysis was conducted to assess the research and development (R&D) costs related to the creation of the *SMART Track* app. This included both the costs of developing the technology and the research costs (mainly time) that were integral to the development of the app, such as workshops to assess development and testing. Furthermore, an estimate of the total time spent in meetings across the R&D process was estimated, from steering group meetings to the conduct of focus groups. The number of hours were estimated to provide additional context of the time invested in the R&D process in developing a comprehensive and user-friendly app.

#### Acceptability Indicators

##### Nested Qualitative Evaluation

The qualitative analysis component of the study was undertaken through two processes: first, as a thematic study to provide insights into the acceptability of the app and the meetings more broadly, which was described in detail in a previous paper [63], and second, as part of the nested evaluation process [98], where the qualitative data were used to support app development and contribute to the experimental nature of the study. Unlike strictly triangulated studies, nested research studies use a combination of data to enrich insights and provide points of comparison to generate new hypotheses [98]. Research questions and interview topics informed the first more deductive coding frame. Categories were summarized and presented with pertinent quotes to the broader team for discussion, which continued until consensus was reached. The analysis sought to shed light on specific questions about the feasibility and acceptability of the app from the perspective of end users. We also explored accounts of the experience of submitting ROMs. Although thematic saturation was not the aim of this part of the analysis, we noticed recurring themes in 12 interviews.

##### Quality Ratings and Digital Therapeutic Alliance

The MARS–User Version (uMARS) [88] and DWAI [92] domain as well as overall mean scores were calculated at the 2-week follow-up.

#### Preliminary Outcomes

Paired sample two-tailed *t* tests were used to compare participant-reported outcomes on the SDS, K-10, and SURE between baseline and the 8-week follow-up.

## Results

### Sample Characteristics

A total of 72 participants were enrolled in this study (Figure 1). The participant characteristics were comparable with prior accounts of SRAU group characteristics [99]; the average age of the participants was 44 years (SD 11), with more men (44/72, 61%) than women (28/72, 39%; Table 3). Most of the participants were born in Australia (59/72, 81%), and 6% (4/72) reported being of Aboriginal, Torres Strait Islander, or both Aboriginal and Torres Strait Islander descent. Employment was the main source of income for almost half of the participants (35/72, 48%).

At baseline, the participants reported attending an average of 6.63 (SD 5.44) SMART Recovery meetings in the preceding 12 weeks (range 0-24). Excessive alcohol consumption was the most common primary behavior of concern, endorsed by 68% (49/72) of participants over the preceding 4 weeks. Injecting drug use (ever) was reported by 25% (18/72), and 11% (8/72) of the sample reported a recent arrest (past 3 months).

**Table 3:** Participant characteristics (n=72).

Variables			Values
Age (years), mean (SD)			44 (11)
**Gender, n (%)**			
	Male	44 (61)	
	Female	28 (39)	
Born in Australia, n (%)			59 (81)^a^
Aboriginal, Torres Strait, or both Aboriginal and Torres Strait Islander descent, n (%)			4 (6)
**Primary source of income^b^, n (%)**			
	Employment (full-time, part-time, or self-employed)	35 (48)	
	Temporary benefit (eg, unemployment)	10 (13)	
	Pension (eg, aged and disability)	13 (18)	
	Other (eg, retirement fund, savings, and investment)	7 (9)	
	No income or dependent on others	5 (6)	
**Highest completed level of education or training, n (%)**			
	High school or less	19 (26)	
	Certificate, diploma, or trade	26 (36)	
	Bachelor’s degree	16 (22)	
	Postgraduate degree	11 (15)	
**Usual accommodation^b^, n (%)**			
	Own home	33 (45)	
	Rented home (public or private)	33 (45)	
	Other (eg, friends, family, and rehab)	4 (5)	
**Marital status^a^, n (%)**			
	Single or unmarried	27 (38)	
	Married or defacto	28 (39)	
	Separated	7 (9)	
	Divorced	8 (11)	
	Widow or widower	1 (1)	
Ever received treatment for a mental health problem			54 (75)
**Self-reported diagnosis received, n (%)**			
	Depression	9 (12)	
	Anxiety	8 (11)	
	Depression and anxiety	22 (30)	
	Other (eg, posttraumatic stress disorder, bipolar disorder, borderline personality disorder, and schizophrenia)	15 (20)	
**Addictive behavior causing the greatest concern, n (%)**			
	Alcohol	49 (68)	
	Amphetamines	7 (9)	
	Cannabis	6 (8)	
	Another drug (eg, cocaine, ecstasy, γ hydroxybutyrate, benzodiazepines)	5 (6)	
	Another behavior (eg, gambling and food)	5 (6)	
**Injecting drug use, n (%)**			
	Within the last 3 months	3 (4)	
	More than three but less than 12 months ago	3 (4)	
	12 months ago or more	10 (13)	
	Never injected	54 (75)	
Arrested in the last 3 months?^b^, n (%)			8 (11)
Overdose (any drug) in the last 3 months^b^, n (%)			1 (1)
**Service use (preceding 3 months), n (%)**			
	Detoxification or withdrawal management	11 (15)	
	Residential rehabilitation	4 (5)	
	Alcohol or other clinic	13 (18)	
	Psychiatrist	13 (18)	
	General practitioner	47 (65)	
	Psychologist	30 (41)	
	Other allied health care provider (nurse, social worker, or counsellor)	17 (23)	
	SMART Recovery	69 (95)^c^	
	12-step	13 (18)	
**Source of referral to SMART Recovery, n (%)**			
	Self	23 (31)	
	Alcohol and/ or other clinic treatment service	17 (23)	
	Mental health care service	13 (18)	
	Legally recommended or mandated	9 (12)	
	Family member or friend	4 (5)	
	Other health care provider or service	6 (8)	

^a^Missing data for 1 participant.

^b^Missing data for 2 participants.

^c^Three new SMART Recovery participants reported that their first meeting (scheduled for the week before baseline assessment) had been canceled. These participants were due to participate in their first group the week of the baseline assessment.

### Feasibility Indicators

#### Enrollment and Engagement

In total, 216 people expressed interest in participating in the study. Of these 216, 97 (44.9%) were deemed eligible, and 72 (33.3%) went on to enroll. Of the 72 participants enrolled in the study, 68 (94%) created an account, 64 (88%) subsequently used *SMART Track* at least once, and 57 (79%) used *SMART Track* multiple times (mean 16.39, SD 16.10; range 2-83 visits). More than half of the participants (42/72, 58%) used *SMART Track* for ≥5 weeks across the study period (Table 4). *SMART Track* was accessed on 74 unique devices (ie, some participants used the app across multiple devices). Apple iPhone (n=33) and Samsung Galaxy (n=21) smartphones were the primary devices used.

**Table 4:** Frequency of SMART Track use expressed as the proportion of study participants per time interval across the 20-week study period (n=72).

	Participants, n (%)
Never	8 (11)
1 week	7 (10)
2-4 weeks	15 (21)
5-8 weeks	25 (35)
>8 weeks	17 (24)

#### Use Patterns

**Figure 3:**
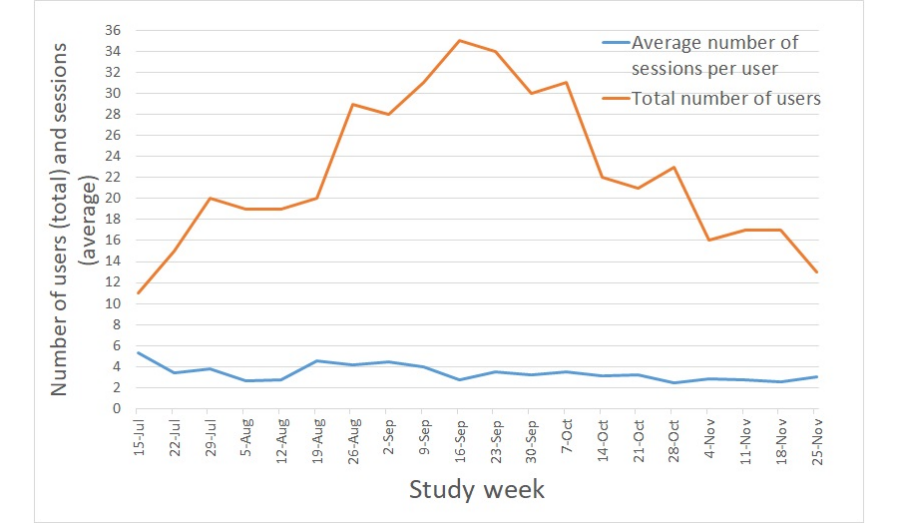
Total number of study participants using SMART Track each week and the corresponding average number of visits per user.

The number of participants using *SMART Track* each week gradually increased across the recruitment period (ie, until the week beginning September 16, 2019), with a gradual decline thereafter (Figure 3). In any one week, the maximum number of study participants using the app was 49% (35/72), and the number of visits to the app ranged from 2.47 to 5.27 (mean 3.39, SD 0.75; Figure 3).

**Figure 4:**
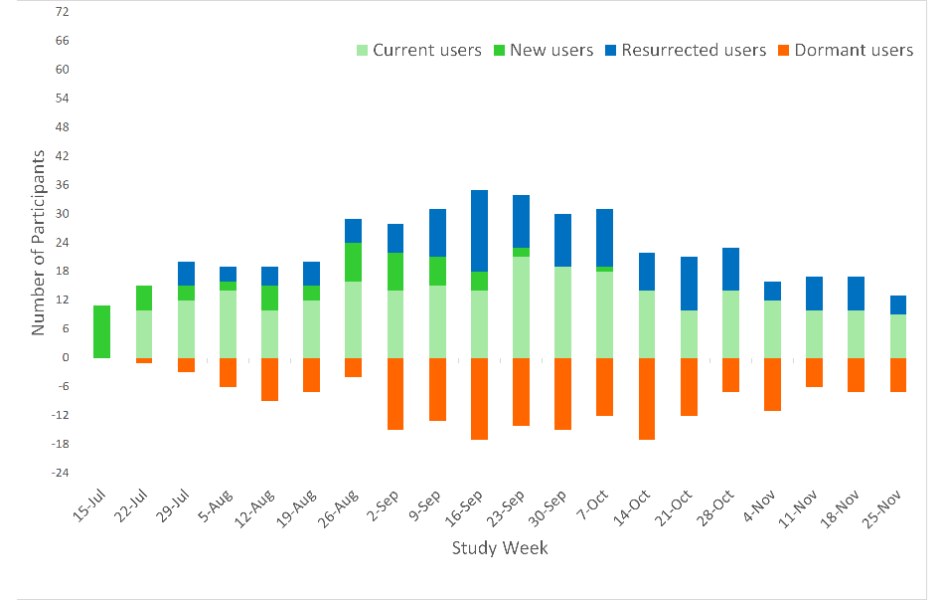
Incoming and outgoing users each week expressed as current versus new versus resurrected versus dormant users.

The changes in the number of new, current, resurrected, and dormant users each week (Figure 4) suggest that the participants typically used *SMART Track* intermittently rather than on a regular (weekly) basis.

The two main *SMART Track* screens designed to provide feedback data (*Urges* screen and *Overall Progress* screen) were the most frequently visited sections of the app (Table 5). The participants spent the most time (minutes) using the *Me* screen and the least time viewing the *Overall Progress* screen (Table 5).

**Table 5:** Use of SMART Track features, expressed as the total number of visits to each of the main screens and the total time spent using each of the main screens.

	Total visits	Total duration (minutes)
Urge button	361	—^a^
Track urges	913	2468.4
Resources	587	443.62
Me	467	3915.94
Overall progress	789	321.33

^a^Duration is not provided for the urge button as use requires a single brief click and is therefore not captured.

Considerably fewer visits were documented for each of the individual progress screens. These sections of the app were visited, on average, only once or twice per week across the duration of the study by a maximum of 8% (6/72) of the participants (Figure S2 of Multimedia Appendix 1).

The study participants most frequently used the app between 6 PM and 9 PM, with almost a quarter of all visits (500/2166, 23.08%) occurring during this time frame. In the morning, use was greatest between 9 AM and midday (Figure S3 of Multimedia Appendix 1).

#### Proportion of Missing ROM Data

**Figure 5:**
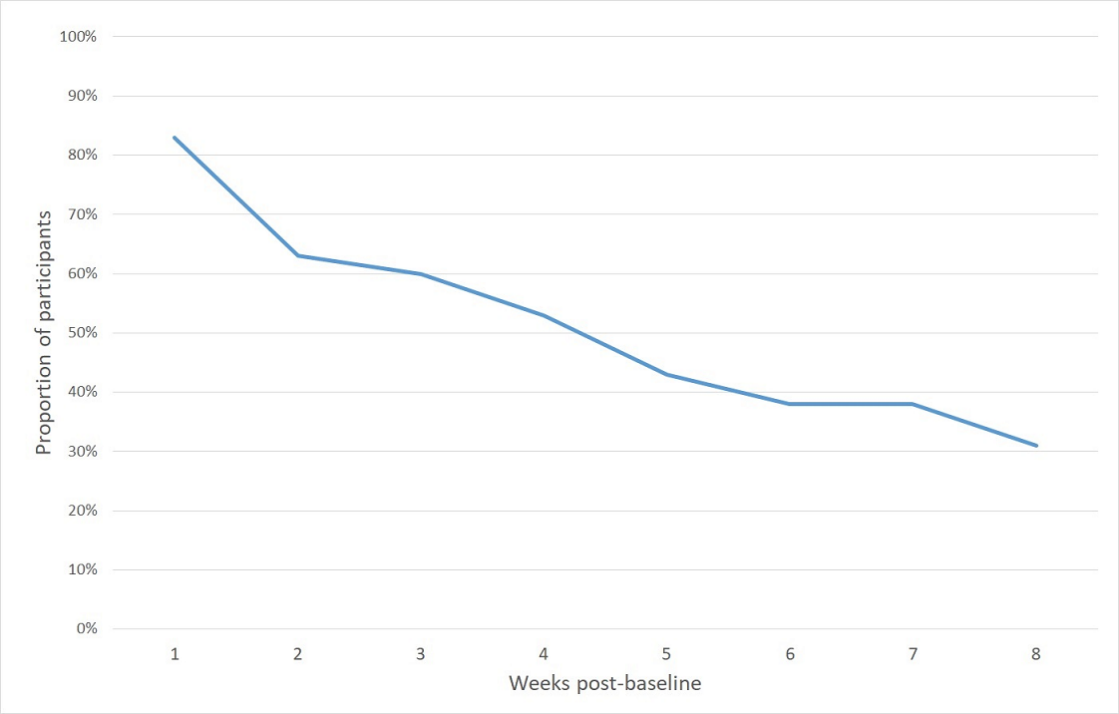
Proportion of study participants using SMART Track to enter routine outcome monitoring data.

During the first week of app use, 83% (60/72) of the participants had used *SMART Track* to enter data for at least one ROM instrument (7-day plan, ROM questionnaire, or urge log). There was a reduction across time in the number of participants completing the ROM items. At the end of 8 weeks, almost a third (22/72, 31%) of the participants had provided ROM data, reflecting a 50% reduction compared with week 1 (Figure 5).

#### R&D Costs

To develop the *SMART Track* app, the developer (GHO) received Aus $76,500. However, the true cost to GHO was more than double (Aus $154,034) when the actual time invested by GHO staff (8 staff members; 876 hours) was fully accounted for (Table 4). Furthermore, the research costs to support the development of the app, such as workshops to assess feasibility and usability testing, were estimated at Aus $127,023 (Table 6). This also includes staff time spent by the trial coordinator and qualitative researcher on development activities. These costs exclude the academic research and evaluation costs that were conducted alongside app development (eg, ethics, recruitment, and economic evaluation; Table 7).

In total, the R&D cost incurred was Aus $203,523. If the true costs to GHO Sydney were included, then the total R&D cost would have been Aus $281,058. Finally, an estimate of the total number of hours invested in the R&D process was estimated at 1485 hours (Table 7). More than three-quarters were in-kind costs and goodwill.

**Table 6:** Cost of SMART Track development and research costs to support development.

Variable			Hours		Rate		Cost (Aus $)
**Development costs**							
	App developer	452		160		72,320	
	Account director	48		180		8640	
	Project manager	73		150		10,875	
	User experience designer	22		180		3870	
	User interface designer	209		180		37,530	
	Strategy director	16		250		4000	
	Creative director	48		350		16,800	
	Total	868		1450		154,035	
**Cost of research to support SMART Track development**							
	Trial coordinator	983		69		67,813	
	SMART Recovery technology lead	224		56		12,617	
	Facilitator support for app	156		58		8992	
	Qualitative researcher	208		144		29,880	
	Transcription (20 interviews, focus group)	N/A^a^		N/A		1759	
	Administrative support	156		38		5962	
	Total	N/A		N/A		127,023	

^a^N/A: not applicable.

**Table 7:** Total hours spent to support the research and development processa.

	Meetings	Duration	People	Person hours
Expert advisory committee	14	45	10	105
Steering committee	2	30	8	10.7
Trial coordinator and external steering committee members	6	30	7	42
Original development company	4	60	7	56
Interviewing new developers	5	5	3	1.3
GHO: preliminary workshops	3	120	15	1080
GHO meetings: design and development	14	60	8	56
GHO: usability testing sessions	9	30	5	22.5
Qualitative researcher meetings	4	60	6	48
SMART Recovery facilitators: focus groups	8	60	8	64
Total	69	500	77	1485.4

^a^Over three-quarters of time was *in-kind*.

### Acceptability Indicators

#### Qualitative Findings

In total, 28 in-depth qualitative interviews were conducted with 20 app users (group members) and 8 facilitators. The participants tended to perceive and describe their use of *SMART Track* within their broader experiences and competencies related to information and communication technology. Of the 20 app users interviewed, the level of knowledge and prior experience of mHealth apps and other digital resources varied widely and were not related to their age or education level. Moreover, the participants’ prior knowledge and experience of apps did not seem to be connected to their use of *SMART Track*. For example, those who demonstrated *high* use of *SMART Track* often described themselves as *new* to apps. Participants with “lots of experience” with apps often described ceasing use after a short time (Alec, group member, low app use).

Compared with another sample of SRAU participants who provided input to inform the development of *SMART Track* [63], the participants in this study expressed less concern about digital support tools replacing face-to-face meetings. These group members tended to perceive the app as complementary to their mutual aid group and described using the resources within it to “stay on track between meetings” (Jasmine, group member, high app use). For these participants, “logging urges and tracking progress” were more desirable than completing routine outcome measures (Campbell, group member, low app use), except when the outcomes data were available to them in “more detail” (Harold, group member, high app use). It is therefore possible that increasing the usability of outcome data tracking activities would increase app users’ engagement with outcome measurements. Consistent with other research, end users seem to be more open to completing repeated and routine outcome measures when their understanding of their outcomes is aligned with the outcome measures selected by the program designers [30,74].

The group facilitator seemed to play a key role in implementing the app and collecting routine outcome data. Facilitators who were knowledgeable about the app and purposefully integrated its use in meetings were more likely to report higher app use among the participants. This is consistent with feedback received from client-participants, who described the facilitators’ efforts, or lack of efforts, when “inspiring” group members to use the app (Mitchell, group member, high app use). Given the lack of experience that some facilitators have with app use and other information and communication technology, it is possible that basic training would have improved their engagement with implementation. In summary, the posttrial interview findings suggest that *SMART Track* is an engaging platform for collecting routine outcome data, and participant concerns expressed at the pretrial time point were not described after the trial.

#### Quality Ratings

The uMARS ratings (Table 8) confirmed the acceptability of *SMART Track*. The overall app quality score was *good*, and every domain was rated as either acceptable or good (ie, uMARS rating >3).

**Table 8:** Quality assessment as indexed by participant responses to the uMARS^a^ and DWAI^b^.

Variable			Values, mean (SD)		Value, median (range)
**uMARS^c^**					
	Engagement	3.6 (0.5)		3.6 (2.0-5.0)	
	Functionality	4.1 (0.7)		4.0 (2.5-5.0)	
	Aesthetics	4.2 (0.6)		4.3 (3.0-5.0)	
	Information	4.3 (0.5)		4.5 (2.5-5.0)	
	Overall quality	4.0 (0.5)		4.1 (3.0-4.9)	
	Subjective quality	3.8 (0.8)		4.0 (1.0-5.0)	
	Perceived impact	3.7 (0.9)		3.7 (1.7-5.0)	
**DWAI^d^**					
	Goals	3.5 (1.0)		3.5 (1.0-5.0)	
	Tasks	3.7 (1.0)		3.5 (2.0-5.0)	
	Bond	3.4 (1.1)		3.5 (1.5-5.0)	
	Overall	3.5 (0.9)		3.7 (1.7-5.0)	

^a^uMARS: Mobile App Rating Scale–User Version.

^b^DWAI: Digital Working Alliance Inventory.

^c^All items are rated on a 5-point scale from 1 (inadequate) to 5 (excellent).

^d^All items are rated on a 5-point scale from 1 (seldom) to 5 (always).

#### Digital Therapeutic Alliance

The DWAI ratings (Table 8) also support the acceptability of the app with the domain scores indicating that, on average, the participants rated the key elements of therapeutic alliance (goals, tasks, and bonds) as being present between *fairly* often and *very* often.

### Preliminary Evidence on Outcomes

There was a significant reduction between baseline and 8-week follow-up for the SDS (mean difference 1.93, SD 3.02; 95% CI 1.12 to 2.73) and K-10 scores (mean difference 3.96, SD 8.31; 95% CI 1.75 to 6.17), but there was no change in the SURE scores (mean difference 0.11, SD 7.97; 95% CI –2.02 to 2.24).

### ROM Reliability

There was a strong relationship between the clinician-administered SURE [74] (at baseline) and the app-administered SURE (week 1; *r*=0.89; *P*<.001). For the quality-of-life item, there was a moderate relationship between baseline clinician administration as part of the EUROHIS-QOL 8-item index [76] and as part of the app-administered measures 2 weeks later (*r*=0.61; *P*=.005). For the Kessler Psychological Distress Scale-6 (K-6) [75], there was a moderate relationship between baseline clinician administration as part of the K-10 [97] and app administration as part of the K-6 2 weeks later (*r*=0.51; *P*=.02). The internal consistency (Cronbach α) for the SURE and K-6 was high across time points and when collected through clinician-interviewed telephone assessments or within the app (SURE: .86 to .94; K-6: .86 to .90).

## Discussion

### Principal Findings

This study was designed to assess the feasibility, acceptability, and preliminary outcomes of *SMART Track* for ROM and feedback in SRAU. The qualitative and quantitative findings support the feasibility, acceptability, and utility of *SMART Track* for ROM and feedback in SRAU. The findings also provide insight into avenues for enhancing sustained engagement. SMART Recovery participants were willing to use *SMART Track*, demonstrated sustained use across the 8-week follow-up interval, engaged most with the two main progress screens (*Urges* and *Overall Progress*), and experienced *SMART Track* as useful and consistent with SMART Recovery principles and strategies. Although it is difficult to attribute it directly to the use of *SMART Track* or SMART Recovery, the participants also showed clinical improvement over the 8-week follow-up, specifically reductions in the severity of dependence and psychological distress. Varied rates of ROM completion, minimal use of domain-specific feedback screens, and qualitative feedback suggest that the utility of *SMART Track* would be improved by making minor changes to app functionality and improving attention to implementation strategies.

### Engagement With SMART Track

To put engagement with *SMART Track* in perspective, it is helpful to consider the rates of engagement with other mHealth apps. One of the challenges with such comparisons is the considerable variation in the metric used to capture mHealth use (eg, mean number of log-ins, sessions, modules, activities completed [100]) and the degree to which these variables are reported [101,102]. Although several systematic reviews of digital recovery support services [39], digital measurement feedback systems [32], and addiction-related mHealth apps are available [54,103-105], the focus tends to be on content, experience, or outcomes, with little to no data examining participant engagement or use. However, the use of mHealth apps by people in recovery from substance use has been shown to vary from as high as 90% in the first few weeks to as low as 18% after 6 weeks [105]. For people with mental health conditions, engagement varied according to the target mental health condition. The number of *nonusers* (individuals who fail to download or use the intervention) has been calculated as 41% (range 25%-58%) for apps targeting depression and 8% (range 0%-16%) for apps targeting anxiety [100]. Reduced engagement over time was common [100]. Compared with these data, participant engagement with, and sustained use of, *SMART Track* is at least comparable, if not higher than the available evidence.

Another useful point of comparison comes from use trends within the global app marketplace. In 2019, data derived from more than 12,000 apps demonstrated that a quarter of the users will abandon an app after one-time use [106]. In comparison, of the 94% (68/72) of the participants in this study who created a *SMART Track* account, more than one occasion of use was documented for 79% (57/72) of the participants. Benchmarks pertaining specifically to lifestyle-related apps (which include fitness-, health-, and travel-related apps) indicate that the average 2-month retention rate is 36% [107]. In comparison, more than half of the participants in this study used *SMART Track* for between 5 and 8 weeks (25/72, 35%) or longer (17/72, 23%), and week 8 ROM data were provided by 31% (22/72) of the study participants.

Uncertainty exists around what is considered a *good* level of mHealth use. Some addiction-related apps (eg, *In My First Year of Recovery* and *A-CHESS* mHealth interventions) have documented high levels of sustained participant engagement (78% program completion and 4-month retention, respectively [105]). In contrast, *SMART Track* use was intermittent, with a proportion of the participants using the app weekly, whereas others disengaged and re-engaged every few weeks. Emerging evidence suggests that engagement with digital recovery support tools may be influenced by recovery duration [39]. Accordingly, engagement with *SMART Track* is likely to vary widely, given that SRAU caters to people across the spectrum of recovery experiences. It is also possible that users may perceive apps as a *short-term commitment* [108]. Therefore, compared with the use of other digital platforms (eg, the web), app use may be shorter and more sporadic [109]. Moreover, training in *SMART Track* was not extensive. The researchers met with the facilitators at each site to orient them to the features of the app. The participants received an email with brief instructions and an in-app onboarding process, although analytics showed that this was used by less than 45.2% (105/232) of users who downloaded *SMART Track* during the study period. The exemplary use rates documented by *In My First Year of Recovery* and *A-CHESS* may have been due to the more active role of counselors in encouraging mHealth use [105] and point to the importance of improved integration of *SMART Track* into SMART Recovery groups. Identifying participant and contextual factors that influence engagement represents an important challenge for future research.

### Routine Outcome Monitoring

A few studies have examined ROM implementation report data on engagement and attrition rates [8,25,28,110,111]. Compared with recent data from the Netherlands, however, the proportion of participants using *SMART Track* to input ROM data is largely comparable, and in some cases, greater than traditional clinician-completed methods [112]. A 50% response rate has been recently suggested as an acceptable benchmark for ROM data and is likely sufficient to protect against bias and yield valid information about patient progress (see study by de Beurs et al [112] for a discussion). In this study, this benchmark was achieved each week across the first month of data collection, with 83% (60/72), 63% (45/72), 60% (43/72), and 53% (38/72) of the study participants completing at least one of the ROM instruments across the first 4 weeks of app use. ROM completion continued to decline during the second month of data collection, with 31% (22/72) of the sample providing ROM data during week 8. Ongoing efforts are needed to improve ROM completion and understand the participant characteristics associated with drop-off (eg, through attrition analyses). For example, given the voluntary, open-enrolling format of SMART Recovery groups and individual variation in group attendance (0-24 in this study), it would be interesting to examine whether app use varies according to group engagement.

### Cost Analysis

The R&D costs of *SMART Track* were estimated at Aus $203,523. However, the developer offered considerable in-kind support, and the overall true cost was Aus $281,058. It is important to reiterate that these are essentially *sunk costs*. If *SMART Track* had been rolled out routinely in SMART Recovery groups, the cost of implementation would have consisted of costs related to app hosting, infrastructure, maintenance, and training. The dynamic nature of the industry makes it difficult to estimate these costs and points to the need for ongoing data collection to understand the long-term real-world feasibility of *SMART Track*.

### Opportunities

To maximize participant engagement in ROM, a clear rationale for why the data are being collected and what they will be used for is essential [25]. The measures must be experienced as *relevant* [110] and the process deemed *worthwhile* [113]. Analytics revealed that few people accessed the detailed, personalized feedback provided within each of the domains listed on the summary page. Qualitative feedback highlighted a mismatch between the effort expended and satisfaction with the feedback provided. Given that the participants were asking for feedback that was already provided in the app (but not accessed), we expect that modifications to improve the visibility of these sections of the app will further enhance ROM completion. Importantly, the regular and frequent use of app self-monitoring features has been linked to a longer period of use and reduced likelihood of abandoning apps [114]. Additional features that have been linked to mHealth engagement include an esthetically pleasing interface, ease of use, degree of personalization, reinforcement (eg, rewards and reminders), communication (with peers or professionals), message presentation (including language, tone, and design), and credibility (encompassing trustworthiness and confidentiality [115]). Although these features were considered throughout the design of *SMART Track* (and positively evaluated as part of the quality assessment), an opportunity exists to further enhance the user experience (eg, through improved personalization, greater use of rewards, and the addition of information-sharing or communication capabilities).

Organizational resources are essential for ensuring the sustained implementation of digital interventions to reduce substance use [105], for example, ensuring leadership support; providing adequate training and resources to both staff and service users; leveraging the expertise of service users to contribute to training; and having a process in place to monitor, evaluate, provide feedback, and respond to uptake rates [116]. Training is a particularly important consideration and provides a forum to (1) build clinician knowledge and confidence in app use and features, (2) practice introducing it to participants, and (3) identify and overcome any concerns or perceived barriers to implementation of mHealth to support routine care [117]. The next steps for *SMART Track* include improved facilitator training and support and leveraging of participant and facilitator *champions* (ie, individuals who actively support the use of *SMART Track*).

### Strengths

The development of *SMART Track* was grounded in theory [40,57] and user-centered design [57]. Consistent with recommendations for enhancing measurement-based care, *SMART Track* includes both standardized and idiographic outcome assessment and harnesses technology to overcome traditional barriers to ROM (eg, scoring and providing tailored feedback [110]). Quality assessment was conducted using a psychometrically valid tool [88], and *SMART Track* surpassed the minimum acceptable quality benchmark (≥3 [88]) on each of the domains assessed by the uMARS, with an overall quality rating of *good*. This is superior to several published accounts [50]. The observed quality of *SMART Track* likely reflects the user-centric approach to development. However, because the ratings were collected as part of a telephone interview with the researcher, the contribution of response bias cannot be ruled out.

Evidence from mental health settings suggests that mHealth apps that have a clear purpose and simple user interface and are easy and time efficient to navigate and demonstrate were more likely to be used as part of routine practice [117]. Quantitative and qualitative data indicate that *SMART Track* possesses these attributes. Pending minor upgrades and improved training and support, this finding further bolsters our confidence in the routine uptake of *SMART Track* within SMART Recovery groups.

### Limitations

This study includes several limitations. In its current format, *SMART Track* is not suitable for people who cannot adequately read and comprehend English. The reliance on written and visual information may also compromise the suitability of *SMART Track* for people with vision impairment. The use of cloud functions for collecting and storing data means that *SMART Track* needs a reliable internet connection to function. The study’s approach to assessing engagement is consistent with recommendations for a multidimensional approach using mobile app data analytics (index of *microengagement*), indices of behavior change (macro level of engagement), and participant subjective experience [102]. However, differences in how various studies define engagement and use make it hard to position *SMART Track* within the context of existing studies. Recent guidelines for the measurement and reporting of engagement data in digital interventions may be beneficial in the future [118].

The current findings are derived from a small sample of participants who attended a limited sample of SRAU groups. We did not collect data on the number of participants attending SRAU groups across the study period; therefore, although participant characteristics are comparable with published accounts [99], generalizability is unclear. Furthermore, the short-term nature of the study makes it challenging to position the findings within the often long-term, nonlinear experience of recovery [16]. Finally, because this is a stage 1 feasibility study, our finding that participants reported reduced severity of dependence and psychological distress from baseline to 8 weeks needs to be interpreted cautiously.

### Conclusions

The qualitative and quantitative findings support the feasibility and acceptability of *SMART Track* and lend insight into avenues for enhancing sustained engagement. Low rates of engagement and high rates of attrition are known challenges for services working with participants who experience substance use and mental health–related difficulties [119]. Sustained engagement with mHealth apps is notoriously difficult to achieve. In light of these challenges, our findings are promising. *SMART Track* offers a potential solution for ROM and feedback, particularly for people with substance use disorders who attend mutual support groups. Future research should focus on identifying relevant demographic, clinical, and contextual factors that may influence the engagement, attrition, and perceived utility of this promising mHealth app.

## Appendix

Multimedia Appendix 1 Supplementary figures.

Multimedia Appendix 2 Supplementary tables.

## Abbreviations

CONSORT: Consolidated Standards of Reporting Trials
DWAI: Digital Working Alliance Inventory
K-10: Kessler Psychological Distress Scale-10
K-6: Kessler Psychological Distress Scale-6
MARS: Mobile App Rating Scale
mHealth: mobile health
R&D: research and development
ROM: routine outcome monitoring
SDS: Severity of Dependence Scale
SRAU: SMART Recovery Australia
SURE: Substance Use Recovery Evaluator
uMARS: Mobile App Rating Scale–User Version
