# Supplementary material for: Feasibility of a Mobile Health App for Routine Outcome Monitoring and Feedback in SMART Recovery Mutual Support Groups: Stage 1 Mixed Methods Pilot Study
Source: J Med Internet Res. 2021 Oct 6;23(10):e25217. doi: 10.2196/25217 (PMC8529481; doi:10.2196/25217)
Supplement: Multimedia Appendix 1 [file jmir_v23i10e25217_app1.docx]

Multimedia Appendix Two:

Supplementary Figures

| 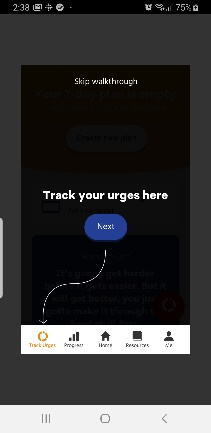 | 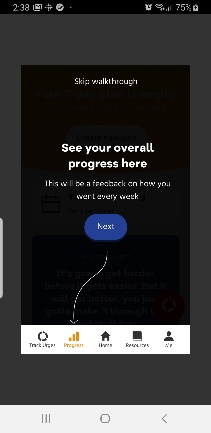 | 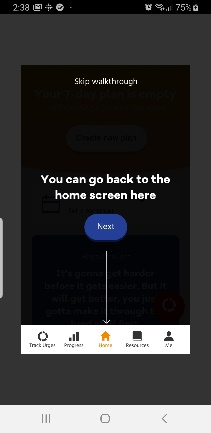 | 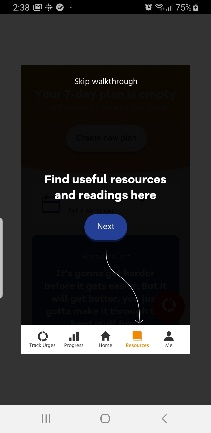 | 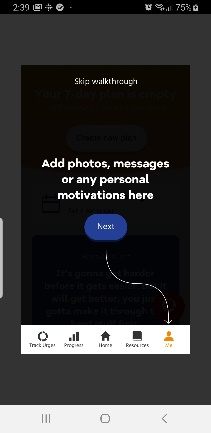 | 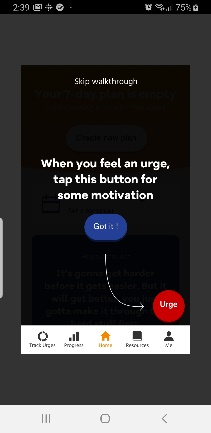 |
| --- | --- | --- | --- | --- | --- |

*Figure S1.*

Participant Walk Through

*Figure S2.*

Total number of visits to the progress screens for each of the individual ROM domains.

*Figure S3.*

Total number of visits to Smart Track presented as the proportion of visits occurring within each three hour interval across the day.
