# Supplementary material for: Feasibility of a Mobile Health App for Routine Outcome Monitoring and Feedback in SMART Recovery Mutual Support Groups: Stage 1 Mixed Methods Pilot Study
Source: J Med Internet Res. 2021 Oct 6;23(10):e25217. doi: 10.2196/25217 (PMC8529481; doi:10.2196/25217)
Supplement: Multimedia Appendix 2 [file jmir_v23i10e25217_app2.docx]

Multimedia Appendix One:

Supplementary Tables

Table S1

Smart Track ROM items and response categories as a function of outcome domain and assessment frequency

| **Domain** | **Frequency** | **Instrument** | **Item(s)** | **Response Option(s)** |
| --- | --- | --- | --- | --- |
| 1. Goal setting | Weekly  (Weeks A and B) | Adapted from the Change Plan  [S1, S2] | Your 7-day plan:  Add task(s) for this week | One or more tasks are added via free text and/ or selecting from some example tasks (e.g. go for a jog, phone a friend), and the participant is prompted to set the time and date for completing each task and whether/ when they wish to set a reminder |
| 1. Values | Weekly  (Weeks A and B)  [per 7-day plan task(s) set] | Adapted from the Valued Living Questionnaire[S3] | This task is important to me for  Family, Intimate relationships, Marriage, Parenting, Friendship, Work, Education, Learning, Recreation, Spirituality, Citizenship, Community involvement, Physical self-care, Psychological self-care, Other | One or more domains are selected |
| 1. Self-efficacy | Weekly  (Weeks A and B) | Confidence Ruler  [S1, S2, S4] | How confident do you feel about achieving this plan? | Sliding scale from 0-10 |
| 1. Goal attainment | Weekly  (Weeks A and B) | Adapted from the Client Rating of Homework Performance[S5] | Your 7-day plan: Task X | For each task, progress is indexed by selecting ‘Done’, ‘Some’ or ‘Not yet started’ |
| 1. Group attendance | Weekly  (Weeks A and B) | --- | Not counting today, how many SMART Recovery groups have you gone to in the last seven days? | 0-7 |
| 1. Frequency of addictive behaviour: Alcohol and other drug use | Weekly  (Weeks A and B) | Adapted from the COMS Questionnaire: Drug and Alcohol Use Scale[S6]^a^ | Which of the following (if any) did you use this week?  Alcohol, Cannabis, Amphetamines, Benzodiazepines, Heroin, Other opioid-based drug, Cigarettes, Other (please specify), None | One or more response options are selected |
|  |  | Adapted from the COMS Questionnaire: Drug and Alcohol Use Scale[S6]^a^ | How many days did you use each of the following? | Sliding scale (0-7) for each of the substances selected at item six |
| 1. Frequency of addictive behaviour: Other addictive behaviours | Weekly  (Weeks A and B) | Adapted from the Screener for Substance and Behavioural Addictions[S7] | Think about the statement “I did it too much”.  In the last seven days, how often did this apply to:  Gambling, Shopping, Sex, Pornography, Internet, Food, Video-gaming, Other | Sliding scale (0-7) for each of the behaviours listed |
| 1. Quantity of alcohol use^b^ | Weekly  (Weeks A and B) | Adapted from the COMS Questionnaire: Drug and Alcohol Use Scale[6]^a^ | On average, how many standard drinks did you have on those days when you were drinking | A number from 0-100 is selected |
| 1. Quantity of cigarette use^c^ | Weekly  (Weeks A and B) | Adapted from the COMS Questionnaire: Drug and Alcohol Use Scale[S6]^a^ | How many cigarettes/cigars/pipes did you have on a typical day when you did use tobacco? _____ cigarettes/cigars/pipes. | A number from 0-100 is selected |
| 1. Alcohol and drug use: Impact | Fortnightly  (Week A) | SURE: Drinking and Drug Use Subscale[S8] | Items 4-6 (e.g. I have coped with problems without misusing drugs or alcohol) | ‘All of the time’, ‘Most of the time’, ‘A fair amount of the time’, ‘A little of the time’, ‘None of the time’ |
| 1. Self-care | Fortnightly  (Week A) | SURE: Self-Care Subscale[S8] | Entire subscale (items 7-11; e.g. I have been taking care of my physical health) | As above |
| 1. Social support | Fortnightly  (Week A) | SURE: Relationships subscale[S8] | Entire subscale (items 12-15; e.g. I have been getting on well with people | As above |
| 1. Resources | Fortnightly  (Week A) | SURE: Material Resources subscale[S8] | Entire subscale (items 16-18; e.g. I have had stable housing) | As above |
| 1. Optimism | Fortnightly  (Week A) | SURE: Outlook on Life subscale[S8] | Entire subscale (items 19-21; e.g. I have felt positive) | As above |
| 1. Mental health | Fortnightly  (Week B) | Kessler – Six Item Questionnaire[S9] | Entire questionnaire | ‘None of the time’, ‘A little of the time’, ‘Some of the time’, ‘Most of the time’, ‘All of the time’ |
| 1. Quality of life | Fortnightly  (Week B) | EUROHIS-QOL 8-item index[S10] | Item One:  How would you rate your quality of life? | ‘Very Good’, ‘Good’, ‘Neither poor nor good’, ‘Poor’, ‘Very Poor’ |
| 1. Urges | As needed | Adapted from The Urge Log ([S1, S2]) | --- | Date and time are automatically captured when the participant clicks on the ‘Urge’ button |
|  |  |  | How intense is your urge? | ‘Low’, ‘Medium’, ‘Strong’ |
|  |  |  | What was going on? (e.g. think about where you were, who was there, what was around you and what you were doing) | Open text |
|  |  |  | How were you feeling?  Tired, Stressed, Hungry, Bored, Upset, Lonely, Relaxed, Happy, Excited, Angry, Worried, Frustrated, Energized, Ashamed, Other | Select one or more of the experiences listed |
|  |  |  | Were you happy with how you handled it? | Yes/ No |
|  |  |  | If yes: What worked for you this time? | Free text |
|  |  |  | If no: What would you like to do differently next time? | Free text |

*Notes^.^* COMS: Client Outcome Management System[S11]; SURE: Substance Use Recovery Evaluator[S8];

^a^This subscale of the Client Outcome Management System is derived from the Brief Treatment Outcome Measure[S12];

^b^Only if participant endorses ‘alcohol’ use over the preceding one week (item six);

^c^Only if participant endorses ‘cigarette’ use over the preceding one week (item six);

Table S2.

Score ranges used to generate participant feedback during week one and two of ROM completion

|  | **7-day Plan**^a^ | **Behaviour of Concern**^b^ | **Effect of Substance Use**^b^ | **Self-Care**^b^ | **Relationships**^b^ | **Outlook on Life**^b^ | **Resources**^b^ | **Mental Health**^c^ |
| --- | --- | --- | --- | --- | --- | --- | --- | --- |
| Doing Well | Majority of tasks marked 'done' | Feedback is not segmented into pre-determined categories to allow the individual to determine their own 'success' based on their own goal(s) | 24+ | 19+ | 16+ | 12+ | 12+ | 19+ |
| Good | Majority of tasks marked 'did some' |  | 14 to 23 | 12 to 18 | 9-15 | 7-11 | 7-11 | 14 to 18 |
| Not so Good | Majority of tasks marked 'not done' |  | < 13 | < 11 | < 8 | < 6 | < 6 | <13 |

**^a^** This content was not ultimately included in Smart Track due to an oversight during Beta testing

^b^ The final scoring metric for the SURE collapses the lowest two (none of the time/ a little of the time) and the highest two (all of the time/ most of the time) response categories and assigns a score of 1-3. To ensure that participants received tailored feedback that acknowledged any level of change (e.g. change from none of the time to a little of the time) the original scoring metric[8] was employed (1-5).

^c^Items were reverse scored to allow high scores to reflect ‘good’ progress, thereby aligning the direction of the mental health graph to the other feedback domains

Table S3. Feedback content according to score range and feedback domain

| **ROM Week** | **Score Range** | **7-day Plan^a^** | **Behaviour of Concern** | **Effect of Substance Use** | **Self-Care** | **Relationships** | **Outlook on Life** | **Resources** | **Mental Health** |
| --- | --- | --- | --- | --- | --- | --- | --- | --- | --- |
| **1** | **Doing Well** | Doing things matter to you and noticing your success is so important - Keep up the great work! | How do you feel about your behaviour this week? How does it fit with what you want to achieve? | Living without too much drinking or drug use shows real progress. What has helped? | When you look after yourself physically and mentally, other aspects of your life also tend to improve. What's it like to notice how well you've been looking after yourself? | Having good relationships is critical to recovery. Keep looking out for others and they will tend to look out for you. | Feeling positive and happy is rewarding. How do you make sure that you keep noticing the good things? | Having some financial or housing stability are good building blocks for getting other aspects of life on track. What's been working well for you? | Not administered |
|  | **Good** | Even if you feel there is more to do - its important to stop and notice what you have achieved - congratulations! |  | Every day without too much drinking or drug use is a step in the right direction. | No middle range | No middle range | No middle range | No middle range | Not administered |
|  | **Not so Good** | Even if it didn't work out as expected - just taking the time to set a goal and think about your progress was a really useful and important thing - good luck next week! |  | Are worried about your drinking or drug use? If so, you might like to chat to your group or someone else you trust. | Are you paying enough attention to your health? Thinking about your health, nutrition, sleep and daily routine - what works for you? | Sometimes it can feel like people around you don't understand you or that there is nobody you can turn to. Where have you gone for support in the past? | Everyone gets down from time to time. Try to think of things you enjoy doing and people you would like to spend time with. It might help to do one or two enjoyable things - no matter how small. | How do you feel about your housing and finances? If things are tough, maybe have a chat to your group or check out the resources page - alcohol and drug services will often be able to provide you with advice on housing or financial problems. | Not administered |
| **2** | **Doing Well** | Nicely done - setting and achieving meaningful goals is such an important part of wellbeing | What's been helping you to change your behaviour? Has anything been getting in the way? | Not administered | Not administered | Not administered | Not administered | Not administered | What's it like to notice that your mental health is in a good place? What has helped? |
|  | **Good** | Great work - we all have weeks where we don’t get through everything we set out to achieve - notice the steps you have taken and see if there's anything you would like to do differently. |  | Not administered | Not administered | Not administered | Not administered | Not administered | How do you feel about your mental health? What have you been doing to look after yourself? Are there any changes you would like to see? |
|  | **Not so Good** | Some weeks are harder than others. Just taking the time to think about what you would like to achieve and how you went is so important. Thanks for checking in. |  | Not administered | Not administered | Not administered | Not administered | Not administered | How do you feel about your mental health? If you would like further information or support, you might like to visit the 'resources' section - or have a chat to your group |
| **3** | **Forward** | Reaching goals and making progress is rewarding. To help you stay on track and keep up your motivation you may like to use the **'Maintaining Motivation'** tool to review the positive changes you have made and the benefits you have gained along the way | What has been going well with your behaviour(s) of concern? What has been going less well? | What's it like to notice that alcohol and other drugs are having less of an impact on you and the way you live your life? What are the most important benefits you have seen? | Looking after yourself physically and mentally is an important part of recovery. What helpful things do you want to keep doing to look after yourself? | What have you been doing to look after your relationships? What's it like to know that you have people around you who you can trust and rely upon? | It can be really rewarding to work towards realistic goals and notice our achievements. What’s it like to notice the things that have been going well? | When accommodation and finances are tracking well, other parts of your life also tend to feel better. What's it like for you? | Not administered |
|  | **Same** | Reaching goals and making progress is rewarding. You may like to use **'The Lifestyle Audit'** exercise to think about what sorts of things you may still like to change – so that you can keep on living the kind of life that works best for you |  | How do you feel about your alcohol and/ or drug use? Are there any changes you would like to make? | How have you been looking after your physical and mental health? What do you want to keep up? Does anything need to change? | How do you feel about your relationships? What do the people around you bring to your life? What do you bring to theirs? | What good things (no matter how small) have you noticed? Keep setting and working towards realistic plans - notice the wins and allow yourself to learn from the challenges. | How do you feel about your living situation and income? What are you grateful for? Are there any changes you would like to see? | Not administered |
|  | **Backward** | If you are feeling overwhelmed and/ or lacking confidence, breaking down change into smaller steps can help. You may like to have a go at making a **Change Plan**. |  | Changing behaviours can be hard, particularly if they’ve been around for a long time. What's worked for you before and how might that help you now? | We all have good weeks and less good weeks - If you were completely successful in looking after yourself, how would things be different? What might be a good next step? | All relationships have their ups and downs. What do you think needs to change? Who could offer helpful support? | When things are tough, it can be helpful to think about what was it like when things were going better for you - How might you be able to do things differently now? | If you’ve been struggling with a situation, the **Problem-Solving** activity may help you to work out your ideas and come up with a plan to help you achieve your goals. | Not administered |
| **4** | **Forward** | Finding enjoyable, meaningful activities and setting goals for your life are an important part of wellbeing. To help you maintain your progress, you may find the **'Lifestyle Balance'** activity useful. | What's it like to see how your behaviour(s) of concern are tracking? What successes have you had? If you need to make a change - what gives you the confidence that you could? | Not administered | Not administered | Not administered | Not administered | Not administered | When your mental health is on-track, what's that like for you? How do you keep things on track - what works best for you? |
|  | **Same** | Finding enjoyable, meaningful activities and setting goals for your life are an important part of wellbeing. The 'Lifestyle Balance' activity may help you to think about the areas of your life that matter to you and the steps you would like to take. |  | Not administered | Not administered | Not administered | Not administered | Not administered | How have you been looking after your mental health? Are there any changes you would like to see? |
|  | **Backward** | Changing the way we set our goals can make it more likely that we will achieve them. You may find the **'Setting SMART Goals'** activity useful to think about the changes you would like to see and how best to get there |  | Not administered | Not administered | Not administered | Not administered | Not administered | Are you worried about your mental health? You might like to chat to your GP about the support options available |
| **5** | **Forward** | The more that you are able to achieve a balanced lifestyle and set goals for yourself, the less likely you are to slip and/ or relapse. The **'Planning an Enjoyable Life'** activity can help you identify the areas you would like to keep working on so that you are living the kind of life that works best for you. | Thinking about the changes you have seen - What skills, resources and strengths have been helpful? [Also have the 'this week vs last week' style feedback?? (Similar to how you did the urge feedback?)] | Changing your relationship to drugs and alcohol can be really rewarding. What gives you confidence that you can keep up the changes you have seen? | Life feels better when we're looking after ourself well - How do you maintain the improvements you have seen? | Giving and receiving support is an important part of recovery for many people. What's it like for you? | Paying attention to the things that are going well can give us confidence to work on things that might be going less well. What are you grateful for? | Having some stability in housing and finances is really helpful - what progress have you already made and how do you keep planning for the future? | Not administered |
|  | **Same** | The more that you are able to achieve a balanced lifestyle and set goals for yourself, the less likely you are to slip and/ or relapse. The 'Planning an Enjoyable Life' activity can help you identify the areas you would like to work on so that you are living the kind of life that works best for you. |  | What helpful things do you want to keep doing? Is there anything you would like to change about your alcohol and drug use? | The process of recovery can feel like a journey. Looking after yourself physically and mentally can be really helpful - what works best for you? | Continue spending time with people you enjoy. Who matters to you? What is meaningful about the relationships in your life? | What things bring enjoyment, meaning and/ or satisfaction to your life? Is there anything you would like to do differently? | Keep on doing those things that keep you in a good space financially and accommodation wise. If there's any other changes you would like to make, maybe have a think about what the next step would be. | Not administered |
|  | **Backward** | Having a clearer idea about where we're going and what we want to achieve can help us to achieve our goals. You might find it useful to give a go at completing the **'Planning & Goal Setting'** activity to help you think about your short, medium and long term goals and how best to achieve them. |  | Your recovery journey will have ups and downs. There are many services and people there to help you. These include your GP, specialist drug and alcohol services, and also your peers in recovery. If you need help - don't be afraid to ask. | What changes have you made in your life that were difficult for you? How did you go about that? What might you be able to do now? | How would you like things to be different? What's helped in the past? What's the next step? | Suppose you did succeed in making the change(s) you want - imagine yourself one year from now - what encouragement would you offer yourself? | Have you been having some challenges with your housing or finances? If you feel that you need some help - don't be afraid to ask. Maybe have a look through the resources page or talk it through with someone you trust. | Not administered |
| **6** | **Forward** | Great work! What's it like to see the positive changes you have made and the benefits you have gained? | Looking at how your behaviour(s) of concern have changed over time, how does that fit with what you want to achieve? What successes would you like to celebrate? What encouragement would you like to give yourself? | Not administered | Not administered | Not administered | Not administered | Not administered | What have you been doing to keep your mental health on track? What helpful things do you want to make sure that you keep doing? |
|  | **Same** | Nicely done - What's it like to see the positive changes you have made and the benefits you have gained? Where to from here? |  | Not administered | Not administered | Not administered | Not administered | Not administered | What helps you to look after your mental health? Is there anything that gets it the way? Where to from here? |
|  | **Backward** | **ABC Tool** Sometimes our decisions lead to behaviour that’s OK for a bit (e.g. feel better), but then ends up costing us in the longer term (e.g. feel guilty).  If this has been happening for you, you may like to use this activity to take a closer look at your thoughts, feelings and behaviours and how your decisions are/ are not working for you.  People often find that getting to know what sets them off can help them to behave differently. |  | Not administered | Not administered | Not administered | Not administered | Not administered | How do you think things are going with your mental health? Who could offer helpful support? |

^a^This content was not ultimately included in Smart Track due to an oversight during Beta testing

Table S4

Schedule for release of resources and motivational stories

| **Resources** | **1** | **2** | **3** | **4** | **5** | **6** | **7** | **8** |
| --- | --- | --- | --- | --- | --- | --- | --- | --- |
| Planning an enjoyable life | ☑ |  |  |  | ☑ | ☑ | ☑ | ☑ |
| Maintaining motivation | ☑ | ☑ |  |  |  | ☑ | ☑ | ☑ |
| The Lifestyle Audit | ☑ | ☑ | ☑ |  |  |  | ☑ | ☑ |
| Goal setting | ☑ | ☑ | ☑ | ☑ |  |  |  | ☑ |
| Self-acceptance | ☑ | ☑ | ☑ | ☑ | ☑ |  |  |  |
| Mindfulness | ☑ | ☑ | ☑ | ☑ | ☑ |  |  | ☑ |
| Coping with urges and cravings | ☑ | ☑ | ☑ | ☑ | ☑ | ☑ |  |  |
| Making a change plan |  | ☑ | ☑ | ☑ | ☑ | ☑ |  |  |
| Problem Solving |  |  | ☑ | ☑ | ☑ | ☑ | ☑ | ☑ |
| Managing your thoughts, feelings  and behaviours |  |  |  | ☑ | ☑ | ☑ | ☑ | ☑ |
| **Motivational Stories** | **1** | **2** | **3** | **4** | **5** | **6** | **7** | **8** |
| Angelo (Voice): Alcohol | ☑ |  |  |  |  |  |  |  |
| Luke (text): Alcohol | ☑ |  |  |  |  |  |  |  |
| Melanie (video): Speed, ice & other stimulants | ☑ |  |  |  |  |  |  |  |
| Opal (voice): Alcohol |  | ☑ |  |  |  |  |  |  |
| Dylan (text): Alcohol |  | ☑ |  |  |  |  |  |  |
| Lala (video): Cannabis |  | ☑ |  |  |  |  |  |  |
| Angelo (voice): Alcohol |  |  | ☑ |  |  |  |  |  |
| Ben (text): Alcohol |  |  | ☑ |  |  |  |  |  |
| Ethan (video): Ice |  |  | ☑ |  |  |  |  |  |
| Emma (voice): Alcohol |  |  |  | ☑ |  |  |  |  |
| Zoe (video): Ice |  |  |  | ☑ |  |  |  |  |
| Nadia (text): Cannabis |  |  |  | ☑ |  |  |  |  |
| Bill (voice): Alcohol |  |  |  |  | ☑ |  |  |  |
| Ethan (video): Speed, ice & other stimulants |  |  |  |  | ☑ |  |  |  |
| Barry (text): Heroin |  |  |  |  | ☑ |  |  |  |
| Opal (voice): Alcohol |  |  |  |  |  | ☑ |  |  |
| Misja (voice): Heroin |  |  |  |  |  | ☑ |  |  |
| Lala (video): Cannabis |  |  |  |  |  | ☑ |  |  |
| Scott (text): Alcohol |  |  |  |  |  |  | ☑ |  |
| Phoenix (voice): Alcohol & prescription meds |  |  |  |  |  |  | ☑ |  |
| Scarlett (video): Speed, ice & other stimulants |  |  |  |  |  |  | ☑ |  |
| Dylan (text): Alcohol & cannabis |  |  |  |  |  |  |  | ☑ |
| Sean (video): Prescription & over the counter  Drugs |  |  |  |  |  |  |  | ☑ |
| Renee (text): Cannabis & speed, ice & other  stimulants |  |  |  |  |  |  |  | ☑ |

Table S5

Interactive Urge Log Content

| Self-Management Strategies |
| --- |
| - Delay until the craving passes. Most urges come and go within 3-5 minutes |
| - Distract yourself. Shift your attention to something else |
| - Decide. Remind yourself of your reasons for change. Congratulate yourself on your progress! |
| - Drink water. |
| - Read a book |
| - Breathe...in for 3-4 seconds...pause for 1-2 seconds...out for 4-6 seconds ...pause and repeat! |
| - Connect with your senses. What are five things that you can See? Smell? Hear? Taste? Touch? |
| - Spend some time with a pet |
| - Listen to music |
| - Go for a walk |
| - Play a game on your phone |
| - Delay – It will help to avoid a poor decision |
| - Delay – it will strengthen your resolve and your skills |
| Tips and Motivations |
| - As soon as I start to feel an urge I start doing artwork or something else creative |
| - Get out of your head and onto your feet |
| - You are worthwhile |
| - It gets easier |
| - You might be doing it hard - but what you are doing is amazing. |
| - Be proud of yourself – it’s hard what you are doing |
| - You are strong, and when you’ve survived through all the shit your addiction has put you through, you can survive recovery. |
| - It does not matter how slowly you go as long as you do not stop. |
| - It will get better, you just gotta make it through the hard stuff first |

Table S6

Pop Up Motivations and Self-Management Strategies

| **Motivational Statements** |
| --- |
| - You are not alone - Just by using this app you are making a difference to others - Regardless of what you say or how things are going it helps to contribute - When you use the app and give data, that kind of information, that kind of support helps everybody - Others have improved - Set yourself up to succeed - Analyse before you criticise - There’s more to life than partying on the weekends and taking drugs - You can be of service just by listening and “being there” - A dip in your progress doesn’t have to affect your determination, your mindset or your outlook - When things get tough, remember, you’ve got the skills to help yourself through - If you say that you are going to be somewhere, there’s more of a chance of you showing up. - It’s not always a ‘feel-good’ meeting. Sometimes people are going through hard times…everyone might be at different stages of recovery, but that’s OK - Remember – no one at SMART is going to judge you if you’ve used or drank or whatever else you might have had dramas with - We all stuff up, it’s human - Even if you’ve stuffed up, it hasn’t been a waste of time. - See if you can learn from your mistakes and keep moving forward - Everyone is in the same boat, we all have wins and also make mistakes in our lives - The key is acknowledging that you have a problem, no matter if it’s an addiction or a loss or whatever. When you do that you are actually meeting that problem and doing something about it - When things suck try to remember “I am going to beat this, I am better than this” - There’ll be times when you’ll say “Maybe I need to take a short cut and go back to what I did before” but you can always choose to say “no, I am not doing that, that’s just stupid” - You’ve got to believe in yourself and not lie to yourself. - If you lie to yourself then you’re not gaining anything. - When you show up, even when you don’t feel like it, you’re showing yourself and others that you can do it - The way in which you handle these challenges will determine how successful you are in making positive changes. - Beliefs come from your own experience and how you see yourself. Sometimes your thoughts are not real and you may not be aware of this - You can’t change the past but you can plan to make good choices for your future. - Acceptance doesn’t mean giving in or giving up. - You can learn from past mistakes and have a brighter future - Recovery is about progression not perfection |
| **Tips** |
| - Devote time to something you enjoy, like a hobby or seeing loved ones - Make a start by doing something that brings a little meaning or purpose to your day - Write stuff down. Monitor how things are going. Its important. - Planning keeps me accountable to myself - Self-monitoring helps me see what helps, see my progress and in doing so be able to help and fix any problems identified along the way - When I’m thinking I could lapse, it helps to renew my reasons for change - Write down what happens when it happens. Take that to a meeting. - Writing things down helps you stick to stuff and be accountable - When I don’t pay enough attention things can just spiral. - It might not always feel good to think about how things are going, but it helps - It’s important to have things in place to make me feel accountable - When I set a goal I find it helpful to check if it’s realistic - If your goal isn’t realistic then don’t make that part of your plan - If I’ve done the wrong thing by myself, then I'll feel guilty and forgive myself and next day say “you’ll do better” and then that’s it – I’ll leave it be. - Working out why I feel like drinking or using at certain times, helps me target that area - What’s your goal and your motivators? - When you track things, you can see the little wins to help you stay on the right track - Refresh your goal and motivators often so you can make the right decisions. - Talking to people helped put me at ease about all the problems I had going on - It’s been really helpful to meet other people in the same boat and learn other strategies - Writing things down can help you see “I haven’t been doing too badly”, otherwise your memory can start to lie to you when you’re in recovery. - I journal to keep me honest, if I let time pass, I think there’s a risk of glossing over and you know … it loses its value. - My journaling and knowing how many things I did is for me a good metric of how I’m going. - Engaging in different community activities is really helpful - When I started dropping off my interests and other activities bada-bing, a month later, I was drinking again. Journaling helped me to see that pattern. - Do something fun, be productive, spend time with people - When you listen to others, you might find that you can relate and that can help you as well - The more that I am able to contribute, the more it reinforces how far I’ve come - Its important to reflect on your progress when things are going well, as well as when things feel stuffed - If you’ve had a few dramas during the week, it might be good to open up a bit - You could get a lot out of opening up and will probably help someone else in the process - Routine and structure works for me. - Tracking progress is a way of seeing changes and patterns. Things you should be concerned about or celebrate. - It can be tricky because when things are getting better it can be easy to think that you don’t need something, so you drop off and then start going down hill again. - Write things down so you know if you are staying on track. This will help you remember the good stuff - When things are going well you’ve got a lot of really good things to share and that’ll help you to stay where you are - It might not always feel like a great meeting, sometimes people will ask you the hard questions, and even though it might not feel good, its sometimes the things you need to hear - Write a list, mark them off, and then when you look at the list and see at least one thing marked off you know you have done something - Without tracking your progress, how do you know if you’re doing better? - If you don’t know what to do with yourself, see what other people have done and try something, you’ll probably surprise yourself! - Its normal to feel a bit lost. Finding meaning in new activities can help - It can be hard to see change. Regular self-reflection helps to see how far you’ve come. - It’s good to think things through and see how you’ve been dealing with things, whether you’ve been avoiding anything. It gives you something to talk about at the group - Every week is different, so the app might make it easier to keep track of everything you’ve got going on - Reading the science behind addiction helped because then it’s not “poor me, well there is nothing I can do, so I may as well keep drinking”, but now I understand how alcohol works on my mind and my body - Hearing other people’s perspective is always a bit of a, “ah, okay.” - Tracking stuff means that you’ll always have something to share, regardless of whether it has been a ‘good’ or ‘bad’ week - Challenge unhelpful thinking by asking “Do I have any experience that shows that this thought is not completely true all of the time?” - If you’re not going through a good time it can be easy to be negative about everything. Having the app might help you be more objective - Challenge unhelpful thinking by asking “What other way(s) could I look at this situation?” - Challenge unhelpful thinking by asking “If I was giving advice to someone I care about who was thinking this, what might I say?” - Ask yourself – what positives or strengths do you have that you might be ignoring? - Often we spend time trying to change our negative, unpleasant thoughts and feelings rather than accepting them for what they are. - If you are able to tolerate and accept the present moment, then it will help you to make the changes needed in the future. - Sharing my experiences with others helps me “give back” - Sharing the things that have been successful and helpful for me and even the things that haven’t been so helpful lets me “give back” - Helping others reminds me of what I’ve learnt and how far I’ve come… so helping other people helps everybody - “Patience, Practice, Persistence” |

Table S7.

Qualitative Interview Topic Guides

| **Participants** | |
| --- | --- |
| For participants who regularly completed the ROM | - We are interested in your thoughts about the ROM. You seemed to use it regularly. What influenced your decisions about using it? - What was useful about the ROM? - What could have been improved?   *Following the initial answers, the interview will prompt with points such as (depending on what the participant has already discussed):*   - - Some people have commented on how often they were asked to complete it. What did you think?   - What did you think about the topics that it covered?   - Some people have said that it was really useful for them in understanding their progress, others had different opinions. What did you think?   - What advice would you like to give to the people who developed the ROM? |
| For participants who did not regularly complete the ROM | - We are interested in your thoughts about the ROM. You didn’t seem to use it regularly. What influenced your decisions about using it? - What was useful about the ROM? - What could have been improved?   *Following the initial answers, the interview will prompt with points such as (depending on what the participant has already discussed):*   - - Some people have commented on how often they were asked to complete it. What did you think?   - What did you think about the topics that it covered?   - Some people have said that it was really useful for them in understanding their progress, others had different opinions. What did you think?   - What advice would you like to give to the people who developed the ROM? |
| **Facilitators** | |
| For facilitators who had participants who regularly completed the ROM | - We are interested in your thoughts about the ROM. Your group members seemed to use it regularly. What do you think influenced their decisions about using it? - What was useful about the ROM? - What could have been improved?   *Following the initial answers, the interview will prompt with points such as (depending on what the participant has already discussed):*   - Some people have commented on how often they were asked to complete it. What did you think? - What did you think about the topics that it covered? - Some people have said that it was really useful for them in understanding their progress, others had different opinions. What did you think? - What advice would you like to give to the people who developed the ROM? |
| For facilitators who had participants who did not regularly complete the ROM | - We are interested in your thoughts about the ROM. Your group members didn’t seem to use it regularly. What do you think influenced their decisions about using it? - What was useful about the ROM? - What could have been improved?   *Following the initial answers, the interview will prompt with points such as (depending on what the participant has already discussed):*   - Some people have commented on how often they were asked to complete it. What did you think? - What did you think about the topics that it covered? - Some people have said that it was really useful for them in understanding their progress, others had different opinions. What did you think? - What advice would you like to give to the people who developed the ROM? |

References

S1. SMART Recovery Australia. SMART Recovery Facilitator Training Manual: Practical Information and Tools to Help You Facilitate a SMART Recovery Group. Haymarket: SMART Recovery Australia; 2015.

S2. SMART Recovery Australia. SMART Recovery Australia Participants' Manual: Tools and strategies to help you manage addictive behaviours. Wooloomooloo: SMART Recovery Australia; 2016.

S3. Wilson KG, Sandoz EK, Kitchens J, Roberts M. The Valued Living Questionnaire: Defining and Measuring Valued Action within a Behavioral Framework. The Psychological Record. 2010;60(2):249-72.

S4. Miller WR, Rollnick S. Motivational interviewing: Helping people change. 3rd ed. New York: Guildford Press; 2013.

S5. Nikolaos Kazantzis FPDaKRR. Assessment of Homework Completion. In: Nikolaos Kazantzis FPD, Kevin R. Ronan and Luciano L'Abate, editor. Using Homework Assignments in Cognitive Behaviour Therapy. New York: Routledge Taylor & Francis Group; 2005. p. 50-60.

S6. NADA. NGO Drug and Alcohol and Mental Health Information Management Project: Determining the Treatment Outcomes Data Collection Set. Strawberry Hills: NADA; 2009.

S7. Schluter MG, Hodgins DC, Wolfe J, Wild TC. Can one simple questionnaire assess substance-related and behavioural addiction problems? Results of a proposed new screener for community epidemiology. Addict. 2018;113(8):1528-37.

S8. Neale J, Vitoratou S, Finch E, Lennon P, Mitcheson L, Panebianco D, et al. Development and validation of 'SURE': A patient reported outcome measure (PROM) for recovery from drug and alcohol dependence. Drug Alch Dep. 2016;165:159-67.

S9. Kessler RC, Andrews G, Colpe LJ, Hiripi E, Mroczek DK, Normand SL, et al. Short screening scales to monitor population prevalences and trends in non-specific psychological distress. Psychol Med. 2002;32(6):959-76.

S10. Rocha NSd, Power MJ, Bushnell DM, Fleck MP. The EUROHIS-QOL 8-Item Index: Comparative Psychometric Properties to Its Parent WHOQOL-BREF. Value in Health. 2012;15(3):449-57.

S11. Network of Alcohol & Other Drugs Agencies. Using the Client Outcomes Management System (COMS). Sydney: NSW Ministry of Health; 2012.

S12. Lawrinson P, Copeland J, Indig D. The brief treatment outcome measure: opioid maintenance pharmacotherapy (BTOM) manual. Sydney; 2003.
